# High Expression of Wee1 Is Associated with Poor Disease-Free Survival in Malignant Melanoma: Potential for Targeted Therapy

**DOI:** 10.1371/journal.pone.0038254

**Published:** 2012-06-12

**Authors:** Gry Irene Magnussen, Ruth Holm, Elisabeth Emilsen, Anne Katrine Ree Rosnes, Ana Slipicevic, Vivi Ann Flørenes

**Affiliations:** 1 Department of Pathology, The Norwegian Radium Hospital, Oslo, Norway; 2 The Wistar Institute, Philadelphia, Pennsylvania, United States of America; The Moffitt Cancer Center & Research Institute, United States of America

## Abstract

Notoriously resistant malignant melanoma is one of the most increasing forms of cancer worldwide; there is thus a precarious need for new treatment options. The Wee1 kinase is a major regulator of the G_2_/M checkpoint, and halts the cell cycle by adding a negative phosphorylation on CDK1 (Tyr15). Additionally, Wee1 has a function in safeguarding the genome integrity during DNA synthesis. To assess the role of Wee1 in development and progression of malignant melanoma we examined its expression in a panel of paraffin-embedded patient derived tissue of benign nevi and primary- and metastatic melanomas, as well as in agarose-embedded cultured melanocytes. We found that Wee1 expression increased in the direction of malignancy, and showed a strong, positive correlation with known biomarkers involved in cell cycle regulation: Cyclin A (p<0.0001), Ki67 (p<0.0001), Cyclin D3 (p = 0.001), p21^Cip1/WAF1^ (p = 0.003), p53 (p = 0.025). Furthermore, high Wee1 expression was associated with thicker primary tumors (p = 0.001), ulceration (p = 0.005) and poor disease-free survival (p = 0.008). Transfections using siWee1 in metastatic melanoma cell lines; WM239^WTp53^, WM45.1^MUTp53^ and LOX^WTp53^, further support our hypothesis of a tumor promoting role of Wee1 in melanomas. Whereas no effect was observed in LOX cells, transfection with siWee1 led to accumulation of cells in G_1_/S and S phase of the cell cycle in WM239 and WM45.1 cells, respectively. Both latter cell lines displayed DNA damage and induction of apoptosis, in the absence of Wee1, indicating that the effect of silencing Wee1 may not be solely dependent of the p53 status of the cells. Together these results reveal the importance of Wee1 as a prognostic biomarker in melanomas, and indicate a potential role for targeted therapy, alone or in combination with other agents.

## Introduction

Malignant melanoma is the second most increasing form of cancer in Norway, following prostate (men) and lung cancer (women) [1]. Whereas the prognosis is good when detected early, there are no curative treatments once the cancer has spread to distant organs (stage IV). Thus, there is a desperate need for new and more effective treatment options.

The cell cycle is the orderly series of events leading to cell division, and is regulated by the assembly and activation of complexes of CDKs and cyclins, which again triggers the transition between each of the four phases; Gap 1 (G_1_), DNA synthesis (S), Gap 2 (G_2_) and mitosis (M). During these events, DNA damages may arise both as a consequence of normal metabolic activity and due to environmental factors, and division may be arrested/delayed at three major DNA damage checkpoints (G_1_/S, intra- S and G_2_/M) before cell division. The G_1_/S checkpoint is largely controlled by p53, a tumor suppressor protein which function is impaired/lost in the majority of cancers, thus compromising this checkpoint. Hence most cancer cells exposed to DNA-damage rely on the S- and G_2_/M checkpoints for repair to occur. Encountering the S-phase checkpoint, genomic insults cause cells to slow down cell cycle progression rather than being arrested, rendering the G_2_/M checkpoint to ultimately halt the cell cycle progression [2]. Central in regulating the transition between the G_2_ and M phases is Wee1-like protein kinase (Wee1), a tyrosine kinase [3]. Wee1 negatively regulates entry into mitosis by phosphorylating the Tyr15 residue of Cyclin Dependent Kinase 1 (CDK1, also known as CDC2), thus inactivating the CDK1/cyclin B complex and arresting the cell cycle.

In addition to being a key regulator of the G_2_/M checkpoint, Wee1 also plays an active role in stabilizing the genome in the S-phase. By suppressing CDK2 activity during DNA synthesis, Wee1 prevents unscheduled initiation of replication that may potentially lead to DNA lesions [4].

Kinases, such as Wee1, represents potential therapeutic targets, however, their expression varies in different types of tumors. Over-expression of Wee1 has previously been reported in osteosarcoma, glioblastoma and breast cancer [5]–[7]. Under-expression, on the other hand, has been described in non-small-cell lung cancer [8]. Cell lines showing an enhanced level of Wee1 have also been demonstrated to be more sensitive to treatment with siWee1 [6]. Due to many promising *in vitro* results, the Wee1-inhibitor MK1775 have very recently been included in two phase I clinical trials both as mono-therapy and in combination with either 5-fluorouracil [9] or topotecan/cisplatin [10].

In the present study, we demonstrate for the first time that Wee1 is up-regulated in human malignant melanomas as compared to normal melanocytes and benign nevi, and that high expression of Wee1 is associated with poor disease-free survival and markers of increased tumor cell proliferation. Our *in vitro* results further revealed a reduced amount of viable cells, accumulation of cells in G_1_/S or S-phase and double-strand DNA breaks following transfection with siWee1 in both p53 wild-type and mutated melanoma cell lines. Together our results indicate a role of Wee1 in proliferation and genomic stability in malignant melanoma, thus potentially making the kinase an eligible therapeutic target.

## Materials and Methods

### Speciments

Formalin-fixed, paraffin-embedded tissue sections from 108 primary malignant melanomas (75 superficial spreading (SSM) and 33 nodular melanomas (NM)), 23 metastases and 10 benign nevi (7 combined, 2 combined+intradermal and 1 intradermal) were randomly collected from the archives of The Norwegian Radium Hospital and regional hospitals. Clinical follow-up was available for all patients. The Regional Committee for Medical Research Ethics South of Norway (S-06151) and The Social and Health Directorate (06/2733) approved the current study protocol.

### Immunohistochemical analysis

Three-µm sections made from formalin-fixed paraffin embedded tissues were immunostained using the Dako EnVision™Flex+ System (K8012, Dako Glostrup, Denmark). Deparaffinization, rehydration and target retrieval were performed in one operation in a Dako PT-link and EnVision™ Flex target retrieval solution with high pH. To block endogenous peroxidase the sections were treated with Dako EnVision Peroxidase Block for 5 minutes. Sections were incubated with monoclonal Wee1 antibody (B-11, sc-5285, 1∶300, 0.67 µg IgG_1_/mL) from Santa Cruz Biotechnology, Inc.(CA, USA) for 30 minutes. Thereafter, the sections were incubated with Dako EnVision™ FLEX+ mouse linker for 15 minutes followed by incubation with Dako EnVision™ FLEX/HRP for an additional 30 minutes. For visualization of staining, the sections were treated with 3′3-diaminobenzidine tetrahydrochloride (DAB) Chromogen (Dako), counterstained with haematoxylin, dehydrated and mounted from xylol with Richard-Allan Scientific Cyto seal XYL (Thermo scientific, MA, USA). Sections from normal placenta with known expression of Wee1 was used as positive control, whereas negative controls included substitution of monoclonal antibody with mouse myeloma protein of the same subclass and concentration as anti-Wee1. Four semiquantitative classes were used to describe the number of stained tumor cells: absent, 0; <10%, 1; 10–50%, 2; >50%, 3. Staining in cytoplasm and nucleus were evaluated separately. Wee1 expression in more than 10% of the tumor cells was considered as high. The expression pattern of Wee1 was compared to proteins previously examined in our melanoma panel, where high expression has been set as >5%: Cyclin A [11], Ki67 [11], Cyclin D3 [12], Cyclin D1 [12], p27 [13] p21^CIP1/WAF1^ [14] and p53 [15].

### Statistical analysis

Statistical analysis was performed using of SPSS version 18.0 (Chicago, IL). The relationship between the expression level of Wee1 and tumor thickness was evaluated non-parametrically using the Mann-Whitney two sample test. Comparison between Wee1 expression and Ki-67, Cyclin A,- D1,- D3, p21^CIP1/WAF1^, p27^kip1^, p53 as well as SSM and NM, was conducted by the use of chi-square tests. Kaplan-Meier survival estimate was used to evaluate the impact on survival.

### Melanocytes isolation

Normal melanocytes were isolated from human foreskins. Briefly, foreskins derived from circumcisions of newborns were washed with Hanks' balanced salt solution (HBSS) (Invitrogen, Carlsbad, CA). Excess adipose tissues were removed, and the skin specimens were cut into approximately 0.5×0.5 cm^2^ pieces and incubated in 0.48% dispase II (Invitrogen) at 4°C. After 18 hours, the epidermis was manually removed from the dermis, cut and digested in 0.05% trypsin for 5 min in 37°C. The suspensions were diluted in 254CF medium (Invitrogen) and serially filtered through 40 µm cell strainers (Becton Dickinson, Franklin Lakes, NJ). The cells were plated in the T25 flask and cultured until confluence was reached. Differential trypsinization was used for first passage in order to obtain a pure melanocyte culture. Once confluent, 2×10^6^ melanocytes were harvested using EDTA, embedded in 200 µL 1.5% agarose and fixed in 10% neutral buffered formalin for 1 hour, and processed by routine histological methods.

### Cell lines and Growth conditions

The human metastatic melanoma cell lines WM45.1 and WM239 were kindly provided by Dr. Meenhard Herlyn (the Wistar institute, Philadelphia, USA) [16], [17]. The LOX cell line was established from a lymph node biopsy of a melanoma metastasis, at the Norwegian Radium Hospital (Oslo University Hospital, Norway) [18]. All cell lines were maintained in RPMI-1640 medium (LONZA, Verviers, Belgium) supplemented with 5% Fetal Calf Serum (Biochrom, KG, Berlin, Germany) and 2 mM L-glutamine (LONZA, Verviers, Belgium). The cells were grown in monolayer culture at 37°C in humidified conditions containing 5% CO_2_ and 95% air.

### Small interfering RNA (siRNA) transfection

All cell lines were plated out in either 6-well plates (1.5×10^5^ cells/well) or in 96-well plates (5×10^3^ cells/well) 24 hrs in advance of the transfection. The cells were transfected with 10 nM siRNA targeting Wee1 (OligioID; ‘VHS50841’) or RNAi negative control duplexes (Negative Control LOW GC, 12935-200) using Lipofectamine™ RNAiMAX transfection reagents (all reagents from Invitrogen corporation, CA, USA).

### MTS assay

Five thousand cells per well were seeded in 96-well plates and left to attach overnight, before siRNA transfection for the indicated time.

Cell viability was determined using the 3-(4,5-dimethylthiazol-2-yl)-5-(3-carboxymethoxyphenyl)-2-(4-sulfophenyl)-2H-tetrazolium) (MTS) assay (Promega, WI, USA), in which the capacity of the cells to convert MTS salt into a brown formazan product was measured. Absorbance was measured at 490 nm using ASYS UVM340 96-well plate reader.

### Trypan blue dye exclusion test

Cells treated with SiCtr or SiWee1 were harvested using trypsin/EDTA (LONZA), along with medium containing floating cells. After centrifugation, the cell pellet was resuspended in PBS containing trypan blue (Merck, Stockholm, Sweden). Viable (dye excluding) and trypan blue stained dead cells were counted.

### Cell Death Detection ELISA^plus^

Determination of cytoplasmic histone-associated-DNA-fragments was assessed using a commercially available kit (Roche Diagnostic GmbH, Mannheim, Germany), following the manufacturers instructions. The presence of histones in cytoplasm is indicative of apoptosis. The ELISA signal was quantified by measuring the absorbance at 405 nm (reference 495 nm), using ASYS UVM340 96-well plate reader (Fisher Scientific, Oslo, Norway).

### Flow cytometric cell cycle analysis

Cells were harvested by trypzination and washed 1× in PBS. Cell pellets containing approximately 10^6^ cells were re-suspended in 1 mL 70% ice-cold methanol and left to fixate for a minimum of 24 hrs. Fixated cells were washed 1× in PBS, and stained with a solution containing 2 µg/mL Hoechst 33258 in PBS. Flow cytometric analysis was performed using LSR II UV laser (BD biosciences, San Jose, CA).

### Western blot analysis

Cells were harvested using a rubber policeman, washed once in 1×PBS, and then lysed in ice-cold NP-40 Lysis buffer (1% NP-40, 10% glycerol, 20 mM Tris-HCl (pH 7.5), 137 mM NaCl, 100 mM NaF), Aprotenin (0.02 mg/mL), Phosphatase inhibitor cocktail 1 (10 µL/mL), Phosphatase inhibitor cocktail 2 (10 µL/mL), PhenylMethaneSulfonyl Fluoride (PMSF) (1 mM), Leupeptin (0.02 mg/mL), Pepstatin (0.02 mg/mL) and Sodium vanadate (1 mM) (Sigma-Aldrich, St. Louis, MO)). Bradford (Bio-Rad Laboratories AB, Sundbyberg, Sweden) analysis was performed for protein quantification, and 25 µg protein/lane was resolved in SDS polyacrylamide gel electrophoresis (PAGE) and transferred to a PDVF immobilon membrane (Millipore, Bedford, MA). To ensure even loading, filters were stained with naphtholblue black (Sigma-Aldrich) and later re-stained with α-tubulin. The membranes were blocked in 5% non-fat milk in TBST (150 mM NaCl, 25 mM Tris-Cl, (pH 7.5), 0.01% Tween 20), and probed with primary antibodies at 4°C overnight, with gentle agitation. Primary antibodies Caspase 3 (#9662/#9664 (even mix)), Caspase 8 (#9746), Caspase 9 (#9502), Cyclin B1 (#4138S), Cyclin D3 (#2936), p21^CIP1/WAF1^ (#2946), p-p38 Thr180/Tyr182 (#4631) and PARP (#9532), were purchased from Cell Signaling (Beverly, MA). α-tubulin (DMIB) was acquired from Calbiochem (Nottingham, UK), whereas Cyclin A (sc-751), p53 (sc-126) and Wee1 (sc-5285) were obtained from Santa Cruz (Santa Cruz, CA). γ-H2AX (#05-636) and pCDK1^Tyr15^ (ab47594) antibodies were acquired from Millipore and Abcam (Cambridge, England), respectively. Membranes were thereafter washed 3×10 minutes in TBST. Membranes were hybridized with an appropriate secondary antibody (HPR-conjugated anti-rabbit or anti-mouse IgG antibodies (Promega)) for 1 hr at room temperature, with gentle agitation, and then washed in TBST for 3×10 minutes. Protein bands were detected after first incubating the membranes with ECL-plus (GE Healthcare, Chalfont St Gils, UK) for 5 minutes, and then exposing them to X-ray films.

## Results

### Increased expression of Wee1 in melanoma

**Figure 1:**
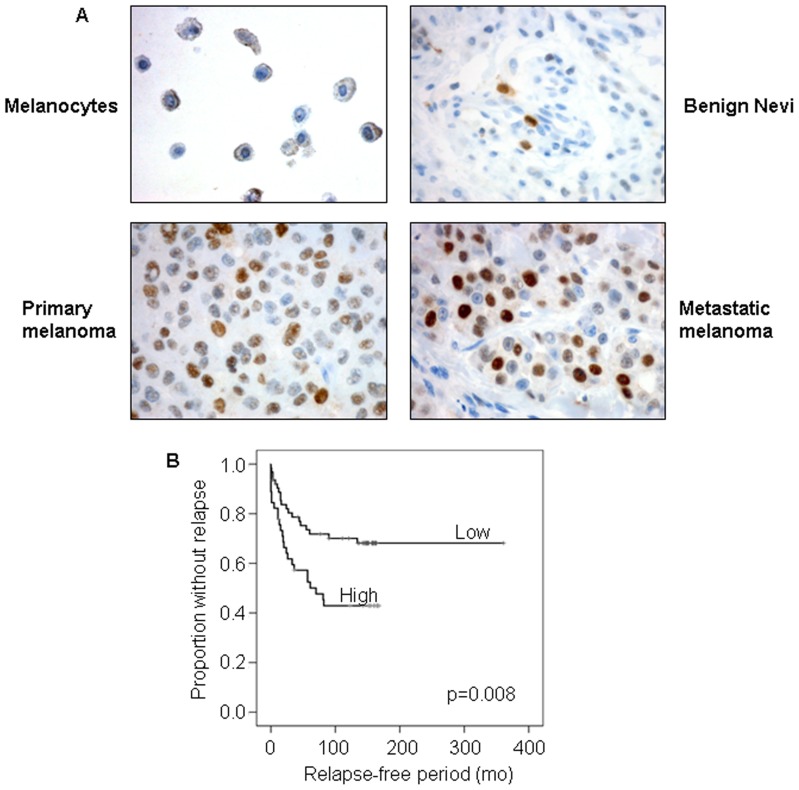
High Wee1 expression increases with tumor progression and is associated with a shorter relapse-free period. A. Wee1 expression in cultured melanocytes, benign nevi, primary- and metastatic melanoma, analyzed by immunohistochemistry. B. Melanoma patients were grouped according to Wee1 expression in their tumors (high (n = 44) or low (n = 63)). Relapse-free survival in months was estimated for both groups and presented as a Kaplan Meyer curve.

High protein expression of Wee1 has previously been reported in human cancers [5]–[7]. Since the status of Wee1 expression in melanomas has not been extensively studied, paraffin-embedded tissue from a panel of benign nevi and primary- and metastatic melanomas, in addition to a sample of cultured melanoncytes, were analyzed for Wee1 protein expression by immunohistochemisty. As illustrated in Figure 1A, protein expression of Wee1 was hardly detectable in the nucleus of the cultured melanocytes, however brown granules were seen in the cytoplasm, most likely due to melanin. Furthermore, as demonstrated in Table 1 and illustrated in Figure 1A, a heterogeneous Wee1 staining pattern was observed in the vast majority of the tumor samples. However, the percentage of positive cells varied in tissues of different stages. Based on distribution, positive immunoreactivity in ≥10% of the tumor cells was used as cut-off to discriminate between high and low Wee1 expression. Whereas only 20% of the nevi displayed Wee1 expression in ≥10% of the tumor cells, this was the case for 42% of the primary- and 70% of the metastatic tumors. Furthermore, while none of the examined nevi contained >50% Wee1 immunoreactive cells, such expression was found in 4% of the primary melanomas and 22% of the metastatic tissues. Nodular lesions expressed higher levels of Wee1 than the superficial spreading tumors. Wee1 expression was in all cases, except two, exclusively localized to the cell nucleus.

**Table 1:** Number (percentage) of melanocytic lesions expressing different levels of Wee1.

Expression level		Low		High	
	No. Analyzed	0%	<10%	10–50%	>50%
Nevi	10	0 (0%)	8 (80%)	2 (20%)	0 (0%)
Primary melanoma	108	3 (3%)	60 (56%)	41 (38%)	4 (4%)
Superficial spreading	75	1 (0%)	49 (65%)	24 (32%)	1 (0%)
Nodular	33	2 (6%)	11 (33%)	17 (52%)	3 (9%)
Metastatic melanoma	23	1 (4%)	6 (26%)	11 (48%)	5 (22%)

Wee1 expressions in benign nevi, primary- and metastatic melanoma were estimated by immunohistochemistry, and categorized in four semi-quantitative classes according to percentage of immunoreactive tumor cells. The groups were further divided into low (<10%) and high (≥10%) expression.

### High expression of Wee1 is associated with poor prognosis and increased proliferation

Since expression of Wee1 increased in direction from nevi to primary- and metastatic melanomas, we next examined the relationship between Wee1 expression, clinical parameters and disease outcome. As shown in Table 2, and Figure 1B, high Wee1 expression (in ≥10% of the tumor cells) was significantly associated with thicker primary tumors (p = 0.001), T-staging (p = 0.004), as well as with ulceration (p = 0.005) and poor disease-free survival (p = 0.008). No association with over-all survival was found (data not shown).

**Table 2:** Wee1 expression correlates with clinical parameters- and markers of tumor progression.

Clinical parameter	No. Analyzed	Expression	Low	High	p-value †
Mean tumor depth	105		1.99 mm	3.90 mm	0.001
T-stage					
T1 (0–1 mm)	27		22 (81%)	5 (19%)	0.004
T2 (1.01–2.0 mm)	34		21 (62%)	13 (38%)	
T3 (2.01–4 mm)	18		7 (39%)	11 (61%)	
T4 (>4 mm)	26		10 (38%)	16 (62%)	
Ulceration	100	No	46 (46%)	22 (22%)	0.005
		Yes	12 (12%)	20 (20%)	
**Marker** *					
Cyclin A	99	Low	39 (39%)	12 (12%)	<0.0001
		High	19 (19%)	29 (29%)	
Ki67	99	Low	46 (46%)	17 (17%)	<0.0001
		High	12 (12%)	24 (24%)	
Cyclin D3	99	Low	48 (48%)	21 (21%)	0.001
		High	10 (10%)	20 (20%)	
p21^Cip1/WAF1^	71	Low	30 (42%)	9 (13%)	0.003
		High	13 (18%)	19 (27%)	
p53	67	Low	38 (57%)	20 (30%)	0.025
		High	2 (3%)	7 (16%)	

* Low expression of Cyclin A [11], Ki67 [11], Cyclin D3 [12], p21 [13] and p53 [14]; defined as immunoreactivity in <5% of the tumor cells. Wee1 expression in <10% of tumor cells is defined as low.

† Statistical significances determined by Chi-square tests.

Since our panel of melanomas has been previously analyzed for other regulators of the cell cycle, we examined the relationship between Wee1 and expression of these parameters (Ki-67, Cyclin A,- D1,- D3, p21^CIP1/WAF1^, p27^kip1^ and p53) [11]–[15]. As shown in Table 2, significant co-variations between Wee1 expression and cyclin A (p<0.0001), Ki67 (p<0.0001), Cyclin D3 (p = 0.001), p53 (p = 0.025) and p21^CIP1/WAF1^ (p = 0.003) were detected. No associations between Wee1 expression and Cyclin D1 and p27^kip1^ were observed (data not shown). Together these results suggest that high Wee1 protein expression is associated with increased proliferation in human melanomas.

### In vitro results support a role of Wee1 in proliferation and genome stabilization

**Figure 2:**
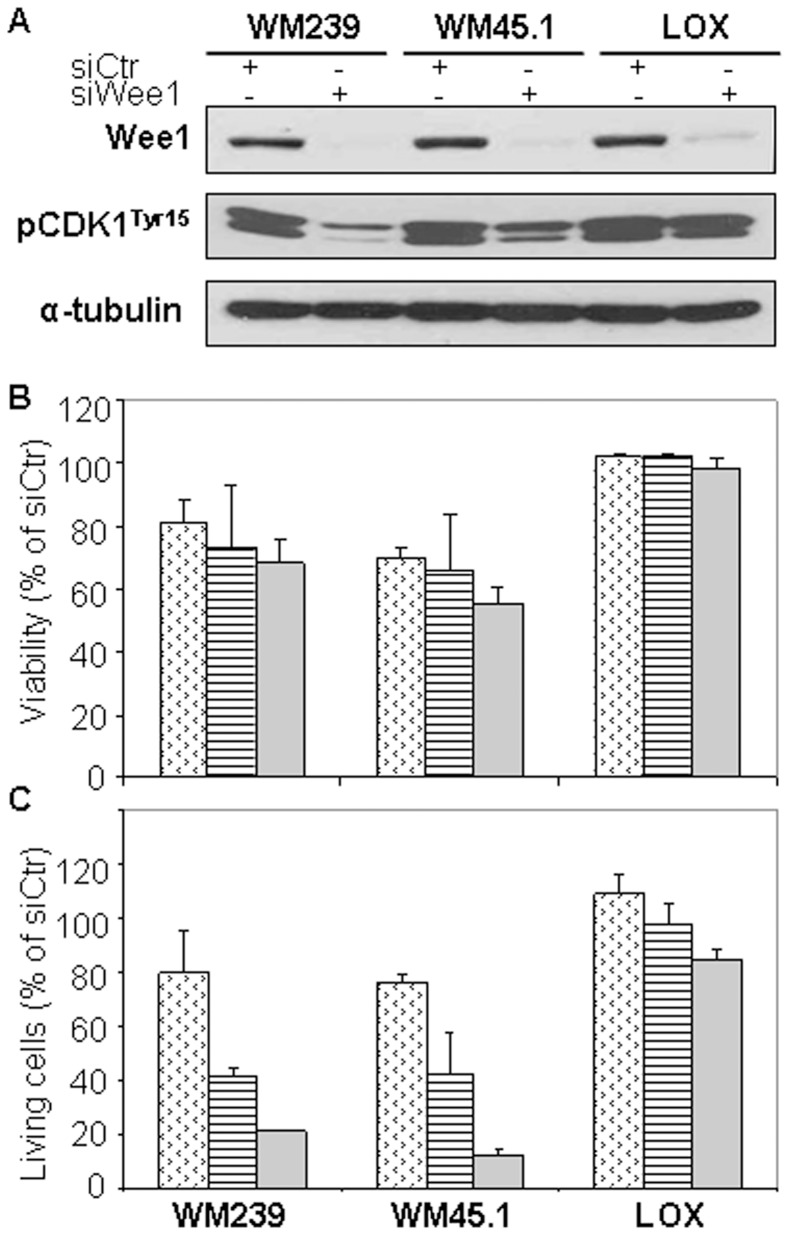
Transfection with siWee1 effectively shuts down Wee1 expression and reduces cell viability. A. Cells were transfected with siWee1 for 48 hours. Expressions of Wee1 and pCDK1^Tyr15^ were examined by western blot analysis. α-tubulin was used as loading control. The figure is representative of at least three independent biological experiments.B and C. Cells were transfected with siWee1 (dots: 24 h, stripes: 48 h and no-pattern: 72 h). The relative amount of viable cells was estimated by MTS (B), and the relative quantity of living cells was estimated by counting trypan-excluding cells (C).

To further study the role of Wee1 in melanomas we knocked-down its expression using siRNA in the three metastatic cell lines, WM239 (p53-wild-type), WM45.1(p53-mutated) and LOX (p53-wild-type). Wee1 was effectively silenced in all three cell lines, as confirmed by western blotting; however, phosphorylation on Tyr15 of CDK1, a downstream target of Wee1, was only down-regulated in WM239 and WM45.1 cells (Figure 2A). Decreased cell viability as estimated by MTS (Figure 2B) and a relative reduction of living cells (Figure 2C), were observed after 24, 48 and 72 hours of siWee1 transfection in WM239 and WM45.1, but not in LOX cells.

**Figure 3:**
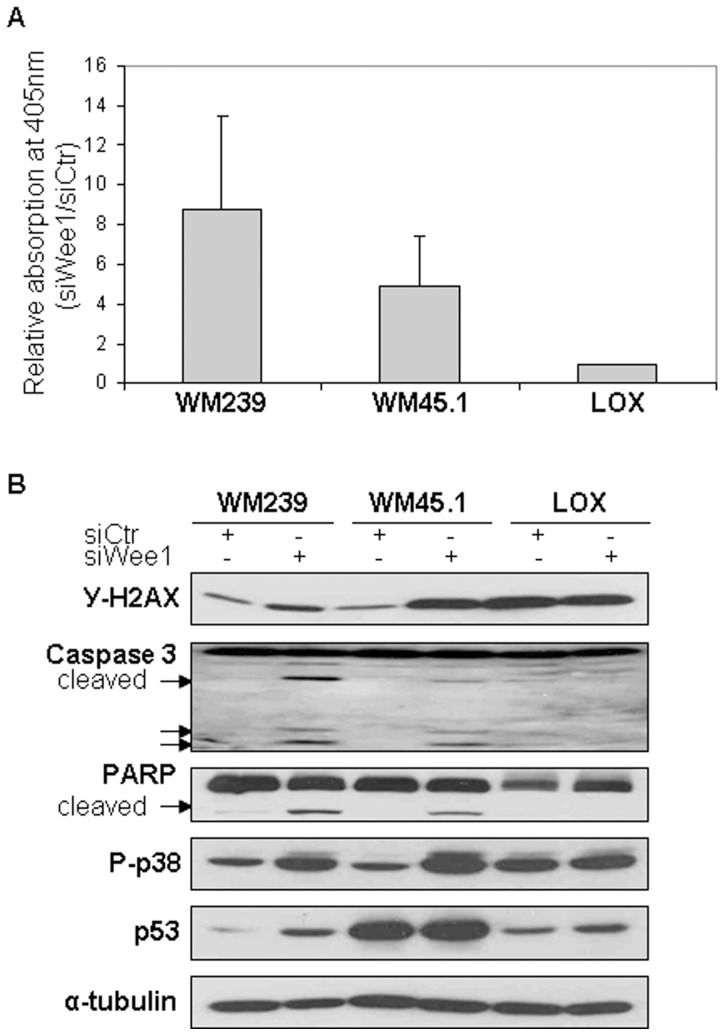
Transfection with siWee1 promotes DNA damages and apoptosis. A. Presence of cytoplasmic oligonucleosomes was measured by ELISA following 48 h siWee1 transfection. Induction of apoptosis shown as enrichment factor calculated as absorbance at 405 nm of siWee1 treated cells relative to siCtr treated cells. B. Protein expressions were measured by Western blot following 48 h transfection with either siCtr or siWee1. Cleavage of PARP and Caspase 3 are shown with arrows. α-tubulin was used as loading control. The figure is representative of at least three independent biological experiments.

Furthermore, we observed that Wee1 silencing led to increased cell death in WM 239 and WM 45.1 as determined by the cell death detection ELISA^plus^ kit (Figure 3A). Likewise, cleavage of Poly(ADP-ribose) polymerase (PARP) and pro-caspase-3, markers of apoptosis [19], were detected in the absence of Wee1 (Figure 3B).

Serine 139 phosphorylation of H2AX (γ-H2AX) is a sensitive marker for DNA double-strand breaks, and may be constitutively expressed in untreated cells due to oxidative DNA damage during metabolic activity [20], [21]. Expression of γ-H2AX was observed in all cell lines, however in the absence of Wee1, an increase of γ-H2AX was observed in WM239 and WM45.1 cells, indicating increased DNA damage (Figure 3B).

The p53 tumor suppressor protein accumulate in the presence of DNA damages, thus leading to DNA repair, cell cycle arrest or apoptosis [22]. An increase in p53 protein expression was observed in WM239 (p53^WT^) cells following treatment with siWee1, but not in WM45.1 (p53^MUT^) or LOX cells (p53^WT^). Notably, these results indicate that inhibition of Wee1 may sensitize melanoma cell lines to DNA damage regardless of their p53 status.

**Figure 4:**
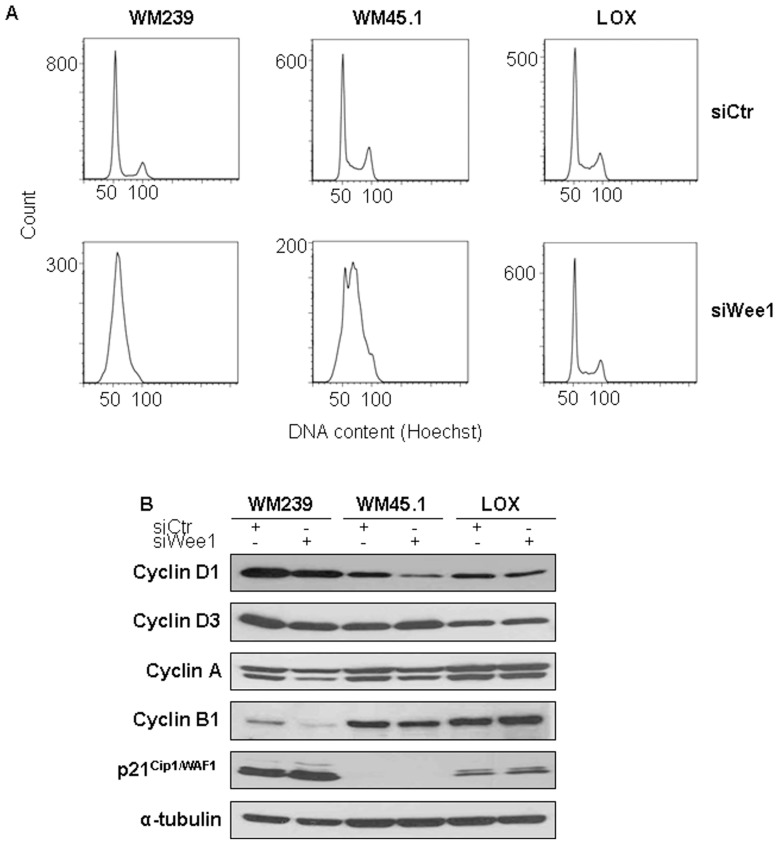
Transfection with siWee1 leads to alterations in cell cycle distribution (A) and -associated proteins (B). A. Histograms showing cell cycle distribution after siWee1 transfection for 48 hours, measured by flow cytometry. B. Cells were treated with siWee1 for 48 hours and protein expressions were analyzed by immunoblotting using the indicated antibodies. α-tubulin was used as loading control. The figure is representative of at least three independent biological experiments.

Since Wee1 is a key regulator of the G_2_/M phase transition, we studied the effect of Wee1 knockdown on cell cycle progression. As demonstrated in Figure 4A, flow cytometry analysis revealed accumulation of WM239 and WM45.1 cells in the G_1_/S- and S-phase, respectively. The cell cycle distribution was, however, not affected in siWee1 treated LOX cells. Furthermore, immunoblotting revealed that cyclin D1, -A, and –B1 protein levels were weakly to moderately down-regulated in WM239 and WM45.1, but not in LOX cells. Moreover, a marginal decrease in cyclin D3 expression was observed in WM239 cells. Despite increased p53 expression, p21^CIP1/WAF1^ protein expression was weakly increased in WM239 cells. No alterations were seen in WM45.1 or LOX cells (Figure 4B). As previously reported, p21^CIP1/WAF1^ was not constitutively expressed in WM45.1 cells, and Wee1 silencing did not affect its expression [23]. The p38 MAP kinase signaling pathway has previously been shown to be involved in p53-independent cell cycle arrest as a response to DNA damage [24], hence we next examined its activation in the absence of Wee1. In support of this hypothesis, increased phosphorylation of p38, indicative of an active signaling pathway, was observed in WM239 and WM45.1, but not in LOX cells, following transfection with siWee1.

## Discussion

In the present study, immunohistochemisty was applied to examine the level of Wee1 in a panel of benign nevi and primary – and metastatic melanomas, as well as in one sample of isolated normal melanocytes, in order to evaluate the impact of altered expression on disease progression and clinical outcome. We demonstrate that Wee1 up-regulation follows tumor progression and is associated with thicker tumors, ulceration and decreased relapse-free survival. Similar results have previously been reported in other forms of human cancers, such as glioblastoma and breast cancer [5], [6]. In non-small-cell lung cancer, on the other hand, reduced Wee1 expression was associated with a higher recurrence rate [8]. Furthermore, Wee1 showed a strong, positive correlation with markers of proliferation: Cyclin A, Ki67 and Cyclin D3 [25]. In support, we have previously reported that increased expression of Ki67, Cyclin A and -D3 is associated with tumor thickness, progression and poor clinical outcome in melanomas [11], [12]. In line with these findings, our *in vitro* results demonstrated that in the absence of Wee1, both Cyclin D1, -D3 (only in WM239) and -A protein expression were weakly decreased in two out of three melanoma cell lines. Based on these findings, we hypothesize that Wee1 contributes to increased proliferation in melanomas.

The augmented expression of Wee1 may seem as a controversy in malignant tumors, based on its well-known inhibitory role in cell cycle progression. However, Wee1 also has a role in genomic stabilization during replication by preventing DNA damage to occur [26], [27]. Furthermore, if other mutations have led to increased CDK- activity, elevated levels of Wee1 may be beneficial to avoid premature mitotic entry resulting in cell death [4]. Our *in vitro* results using siRNA mediated downregulation of Wee1, led to increased cell death, thus further emphasizing the association with malignancy observed *in vivo*. It is therefore likely that the high levels of Wee1 observed protect the melanoma cells against DNA damage and cell death. In line with this hypothesis, our *in vitro* results showed that double-strand DNA damage, as demonstrated by increased γH2AX expression, occurred in the absence of Wee1 in both WM239 and WM45.1 cells, and was accompanied by accumulation of cells in the G_1_/S- and S phases, in the two cell lines, respectively. In accordance with our results, Dominiguez-Kelly *et al.* [27] recently reported augmented amounts of γH2AX in cells stalled in the S-phase, following treatment with siWee1. Given its role in maintaining genomic stability in S-phase, we speculate whether cells lacking Wee1 may fail to regulate CDK activity during replication, thus leading to DNA damage and S-phase arrest [26]. Another possible explanation may be that cells in lack of a functional G_2_/M checkpoint rush into mitosis without securing a proper DNA synthesis, potentially leading to so-called mitotic catastrophe and cell death [7], [28].

In accordance with a study by Hashimoto et al. [29], using Wee1 inhibitor PD0166285 in murine melanoma, silencing of Wee1 also led to decreased proliferation in WM239 and WM45.1 cells in the present study. Strikingly, the growth inhibitory effect in murine melanomas was even stronger than what was observed in our study following siWee1 transfection. However, whereas siWee1 is believed to be highly specific, PD0166585 is a nonselective Wee1 inhibitor which even at low concentrations can target a range of other kinases involved in regulating CDK activity, such as Membrane-associated tyrosine/threonine protein kinase 1 (MYT1) and Serine/threonine-protein kinase 1 (CHK1) [28], [30]. The increased effect may also simply be due to the differences in tumor cell lines. Notably, silencing of Wee1 had no effect on LOX cells in terms of proliferation, cell death or cell cycle distribution. However, phosphorylation of its downstream target CDK1^Tyr15^ was not abolished in this cell line, thereby providing a possible rationale for lack of response to treatment with siWee1. Hence, we speculate if other mechanisms are more central in CDK1 regulation in this cell line, for instance MYT1, known from other cell systems to have much of the same functions as Wee1 [31].

In the present study we found that Wee1 had a strong positive correlation with p53 expression and p21^CIP1/WAF1^ in primary melanomas. High p53 expression has previously been shown to correlate with poor clinical outcome and increased proliferation in metastatic melanoma [32], [33], however the opposite has also been found [34]. Likewise, we have previously reported that p53 protein expression is increased in metastatic melanoma compared to benign nevi, however, although not significant, high expression was also associated with a more favorable disease progression [15]. Despite being mutated in the majority of human cancers, mutational inactivation of p53 is rare in melanomas; yet the protein may not function as normal. In this regard, it was shown that despite being expressed as wild-type in melanoma, p53 could activate some genes in response to stress, but lacked the ability to inhibit growth or induce apoptosis [33], [35]. Interestingly, our *in vitro* results demonstrated that the effects of silencing Wee1 were not exclusive to p53 mutated cell lines. In contrast to our findings, effects of inhibiting Wee1 in other cancer forms have in previous studies been described as limited to cells with mutated p53, in particular when combined with DNA damaging agents [36]–[38].

Additionally, p21^CIP1/WAF1^, a down-stream target of p53, was significantly correlated with Wee1 in primary melanomas. p21^CIP1/WAF1^ is a well-known inhibitor of CDKs, and is known to promote cell-cycle arrest in response to many stimuli, however the protein may also exhibit oncogenic activities [39]. In line with this, we have previously demonstrated that p21^CIP1/WAF1^ expression is up-regulated in primary melanomas compared to benign nevi, and is associated with thicker tumors [14]. When silencing Wee1 *in vitro*, p21^CIP1/WAF1^ expression increased marginally, in WM239 cells only, suggesting that the association between Wee1 and p21^CIP1/WAF1^ observed *in vivo* could be due to indirect mechanisms. However, the accumulation of cells in G_1_/S phase, accompanied by increased p21^CIP1/WAF1^ protein expression, in siWee1 treated WM239^p53wt^ cells, suggests that the augmented p53 protein level probably is able to trigger the G_1_/S checkpoint in response to DNA damage in this cell line. Hence we speculate if the increased cell death seen in WM239 cells in the absence of Wee1, is related to the cells inability to control CDK activity during DNA replication, rather than the ability to stop the cell cycle progression in G_2_/M. Notably, Reinhardt *et al.* has previously reported that activation of the p38/MAPK signaling pathway may cause cell cycle arrest after DNA damage in the absence of p53 [24]. In both WM239 and WM45.1 cells activation of the p38/MAPK signaling pathway increased in the absence of Wee1 suggesting that the p38/MAPK signaling pathway may contribute to the arrest following DNA damages induced by siWee1.

In conclusion, our results indicate that despite being an inhibitor of cell cycle progression, high expression of Wee1 is associated with malignancy and poor prognosis in patients with melanoma. Our *in vitro* results further support these findings; silencing of Wee1 resulted in DNA damage and increased cell death in two out of three cell lines regardless of p53 status. Thus, high expression of Wee1 appears to protect the cancer cell from DNA damage and ultimately cell death. These findings potentially make Wee1 an eligible target in melanoma, both as mono-therapy and in combination with DNA damaging agents.
